# The safety and tolerability of berdazimer gel 10.3% in Japanese patients with molluscum contagiosum

**DOI:** 10.1016/j.jdin.2024.09.002

**Published:** 2024-10-05

**Authors:** Makoto Kawashima, Yoshiyuki Kaneko, Manami Sawasaki, Kyohei Masubuchi, Hiroyuki Yasukawa, Saki Okada, Carolyn Enloe, Carri Geer, Martina Cartwright, Tomoko Maeda-Chubachi, Takeshi Tani

**Affiliations:** a Department of Dermatology, Tokyo Women’s Medical University, Tokyo, Japan; b Sato Pharmaceutical Co., Ltd., Tokyo, Japan; c Pelthos Therapeutics, Durham, North Carolina

**Keywords:** berdazimer gel, Japanese patients, molluscum contagiosum, nitric oxide

## Abstract

**Background:**

Molluscum contagiosum (MC) is a contagious viral skin infection. Berdazimer gel, 10.3% (SB206 12%) is approved in the United States as the first topical, at-home MC prescription medication.

**Objective:**

To assess safety and tolerability of SB206 12% in Japanese patients with MC.

**Methods:**

SKN15B01 (JRCT2031230123) was a phase 2, multicenter, single-group, open-label study in Japanese patients ≥2 years old with 3-70 baseline MC lesions. Patients with only periocular MC and/or immunosuppression were excluded. SB206 12% was applied once daily to lesions for 12 weeks. Safety endpoints included adverse events and local skin reactions. Exploratory efficacy endpoints included percentage of patients with complete lesion clearance and lesion count percent change from baseline.

**Results:**

Twenty patients (12 males, 8 females) with mean (range) age of 5.2 (3-13) years and mean (range) baseline lesion counts of 22.5 (8-44). Complete clearance and percent change from baseline at week 12 were 60% and −89.2%, respectively. The most common treatment-related adverse events were application site-erythema (25%, *n* = 5), -pain (25%, *n* = 5), -pruritis (25%, *n* = 5), and -dermatitis (20%, *n* = 4), all mild/moderate in severity.

**Limitations:**

Single-group study; small sample size.

**Conclusion:**

Safety and efficacy of berdazimer gel, 10.3% in Japanese patients were favorable and consistent with previous studies.

**Capsule Summary**

• The safety and efficacy of US Food and Drug Administration–approved berdazimer gel, 10.3% for the topical treatment of molluscum contagiosum in Japanese patients is unknown.
• Once-daily berdazimer gel treatment in Japanese patients with molluscum contagiosum showed favorable safety, tolerability, and efficacy, consistent with previous studies.

## Introduction

Molluscum contagiosum (MC) is a highly contagious viral skin infection caused by the *Molluscipox* virus (MCV).^1^ There are 4 types of MCV (MCV1, MCV2, MCV3, and MCV4); MCV 1 affects >90% of children with MC, and MCV4 has only been found in Asia and Australia.^1^, ^2^, ^3^ The virus replicates in the epidermis, specifically in the basal layer, where infected cells rapidly proliferate leading to the development of Henderson-Patterson bodies containing MCV virions.^1^ Externally, lesions appear as white or skin-colored papules 2 to 5 mm in diameter with a central indentation. MC lesions can appear anywhere on the body; most people infected with MC will present with 1 to 20 lesions,^4^ yet in severe cases >100 lesions may be seen, particularly in immunocompromised patients.^5^ Lesions may become irritated and itchy; touching or scratching lesions promotes autoinoculation, allowing lesions to spread to other parts of the body.

MC primarily infects children under 14 years of age.^6^ MCV infections are contracted through skin-to-skin contact with infected individuals and contaminated objects like toys, towels, and clothing.^1^^,^^7^

The 2010 Global Burden of Skin Disease places MC in the top 50 most prevalent diseases worldwide.^8^ In 2017, there were an estimated 12,161,039 MC cases in 7 countries with the United States leading in prevalence with about 6,163,809 cases, followed by Japan with approximately 1,584,927 MC cases.^9^ A recent Japanese study reported the cumulative incidence of MC at 8 years of age was 47.1%, with most patients presenting with 30 or fewer lesions.^10^ Curettage is the most frequently used treatment for MC in Japan. As in the US, dermatologists were more likely than pediatricians to remove lesions using this procedure, whereas pediatricians favored no treatment.^10^^,^^11^ MC treatment in Japan is largely influenced by physician preference.^10^

MC may resolve on its own after several months or years, hence treatment is typically not initiated.^1^^,^^4^^,^^5^ However, untreated MC is burdensome and associated with missed school, limited social interactions, stigma, prolonged contagiousness, and time-consuming viral containment activities. In Japan, the only treatment for MC that is covered by health insurance is curettage. However, because curettage is associated with bleeding and pain, there is ongoing controversy as to whether it should be performed in patients with MC, particularly in children.

Recently, 2 MC treatments were approved by the US Food and Drug Administration (FDA). YCanth (0.7% cantharidin, Verrica Pharmaceuticals Inc.), a drug-device combination of a blistering agent that requires application in a healthcare provider’s office and post-application wash off, was approved in 2023.^12^ Zelsuvmi (berdazimer topical gel, 10.3%, also known as SB206 12%, EPIH SPV, LLC), a first-in-class, topical, nitric oxide releasing prescription medication that can be applied by the patient or caregiver once a day and does not require postapplication removal, was approved in 2024.^13^^,^^14^ Neither product is approved in Japan. FDA approval of berdazimer gel, 10.3% was based on 3 phase 3 clinical trials that enrolled 1598 patients with MC in the US who were primarily of European descent.^15^^,^^16^

The mechanism of action of berdazimer gel in the treatment of MC is unknown. However, berdazimer sodium (the active ingredient in berdazimer gel, 10.3% [SB206 12%]), has shown antiviral and immunomodulatory activity in vitro,^17^ providing a plausible mechanistic explanation for the phase 3 efficacy results. Berdazimer gel, 10.3% is supplied as 2 tubes of gel that are mixed on a dosing guide; mixing the gel from each tube causes the release of nitric oxide, and the gel is then applied using a fingertip directly on the MC lesions.

This open-label phase 2 study investigated the safety and tolerability of once-daily application of berdazimer gel, 10.3% in 20 Japanese patients with MC. The efficacy of berdazimer was also assessed in an exploratory manner.

## Methods

SKN15B01 was an open-label, single-group, multicenter, phase 2 study conducted at 5 sites in Japan. The protocol was approved by an institutional review board (4 sites were approved 25 May 2023 and 1 site was approved 8 June 2023) and patients/caregivers provided written or oral informed assent/consent before study treatment was initiated. The authors obtained written consent from patients for their photographs and medical information to be published in print and online and with the understanding that this information may be publicly available. Patient consent forms were not provided to the journal but are retained by the authors. Japanese Registry of Clinical Trials (https://jrct.niph.go.jp): JRCT2031230123 (registered 24 May 2023).

### Patients

Male and female patients ≥2 years of age with 3 to 70 molluscum lesions were eligible. Key exclusion criteria included patients with only periocular MC lesions, and/or immunosuppression.

### Interventions

Patients or their caregivers applied a thin layer of berdazimer gel, 10.3% for up to 12 weeks to all lesions identified at baseline and new lesions appearing anytime during the study. Before the week 4 visit, treatment continued until the next scheduled visit even if the patient believed the lesion(s) cleared. After week 4, patients could discontinue treatment if they believed the lesion(s) cleared. If new lesions appeared or previously cleared lesions recurred after complete clearance, treatment was reinitiated. No study treatments were provided after week 12.

### Assessments

#### Efficacy

Raised and palpable molluscum lesions were counted at weeks 2, 4, 8, and 12. Investigator/Subject Global Severity Assessment (I/S-GSA) were assessed at baseline and week 12 based on the following 5-point scale: 0 = none, 1 = mild, 2 = moderate, 3 = severe, and 4 = very severe. Investigator/Subject Global Impression of Change (I/S-GIC) was assessed at week 12 on the following 7-point scale: 1 = very much improved, 2 = much improved, 3 = minimally improved, 4 = no change, 5 = minimally worse, 6 = much worse, and 7 = very much worse.

#### Safety

Adverse events (AEs) and local skin reactions (LSRs) were evaluated. LSR scoring included assessment of 6 dimensions (erythema, flaking/scaling, crusting, swelling, vesiculation/pustulation, and erosion/ulceration of a representative area), with each dimension individually assessed using a 5-point scale (Table I). Methemoglobin is produced in the body during the reaction of hemoglobin with nitric oxide, and the level was measured by pulse co-oximeter at baseline and week 12 or the first visit when complete clearance was observed. LSRs were assessed at every visit, and if clinically significant as judged by the investigator, they were temporarily reported as AEs. Determining whether a clinically significant LSR was the beginning-of-the-end (BOTE) sign, drug induced irritation (DII), or a mixture of both, at the visit when the LSR is observed, has been difficult in previous studies.^18^ Therefore, the designation was determined retrospectively at week 12, taking into account the course of the patient’s molluscum lesions for this study. LSRs judged as DII or a mixture of BOTE and DII were ultimately treated as AEs. In addition, treated areas were assessed for scarring/keloids. Photos were taken of target areas (ie, an area where as many lesions as possible could be photographed) at baseline and weeks 1, 2, 4, 8, and 12. LSRs reported as moderate or severe in intensity were also photographed whenever possible.

**Table I:** Local skin reaction score∗

	Erythema	Flaking/scaling	Crusting	Swelling	Vesiculation/pustulation	Erosion/ulceration
0	Not present	Not present	Not present	Not present	Not present	Not present
1	Slightly pink	Mild, limited	Isolated crusting	Minimal, limited	Fine vesicles	Superficial erosion
2	Pink or light red	Moderate	Crusting <50%	Mild, palpable	Scant transudate or exudate	Moderate erosion
3	Red, restricted to treatment area	Coarse	Crusting >50%	Moderate	Moderate transudate or exudate	Marked, extensive
4	Red extending outside treatment area	Scaling extending outside treatment area	Crusting extending outside treatment area	Marked swelling extending outside treatment area	Marked transudate or exudate	Black eschar or ulceration

∗ Assessment based on representative lesions.

### Main outcome measures

Safety and tolerability assessments included the number of patients and the number of events for each AE and AE type and severity, LSRs, and methemoglobin levels.

Exploratory efficacy endpoints were: percentage of patients with complete clearance of all treatable MC lesions; percentage of patients achieving at least a 90% reduction from baseline in the number of MC lesions; percentage of patients achieving at least a 75% reduction from baseline in the number of MC lesions; percentage of patients achieving a lesion count of 0 or 1; percent change from baseline in the number of all treatable MC lesions; absolute change from baseline in number of all treatable MC lesions; percentage of patients who had a recurrence of MC; patient-reported spread to household members as measured by any new occurrence of MC in household members. I/S-GSA and I/S-GIC scores were tabulated by frequency.

### Statistical analysis

No formal statistical analyses were planned; therefore, no power calculation was performed. The study used a convenience sample of 20 patients. Data were analyzed descriptively.

## Results

The first patient was enrolled on 07 June 2023 and the last patient completed the trial 12 December 2023. Overall, 20 patients (12 males and 8 females) were enrolled, and all received berdazimer gel, 10.3% once daily for treatment of molluscum lesions. Baseline demographic and clinical characteristics of the enrolled patients are shown in Table II. On average, patients were 5.2 years of age and had a baseline lesion count of 22.5. The majority of patients (65%) did not present with the BOTE sign at baseline.

**Table II:** Baseline demographic and clinical characteristics

Characteristic	Berdazimer gel, 10.3% *N* = 20 *n* (%)
Biological sex	
Male	12 (60.0)
Female	8 (40.0)
Age, years, mean SD [range]	5.2 (2.3) [3-13]
Age group, years, *n* (%)	
≥2 to <6	13 (65.0)
≥6 to <12	6 (30.0)
≥12 to <18	1 (5.0)
≥18	0
Baseline lesion count, mean (SD) [range]	22.5 (9.6) [8-44]
Baseline BOTE sign, *n* (%)	
Yes	7 (35.0)
No	13 (65.0)
Months since onset of symptoms of current episode, mean (SD) [range]	5.6 (6.6) [0.36-31.1]

*BOTE*, Beginning-of-the-end; *SD*, standard deviation.

**Fig 1:**
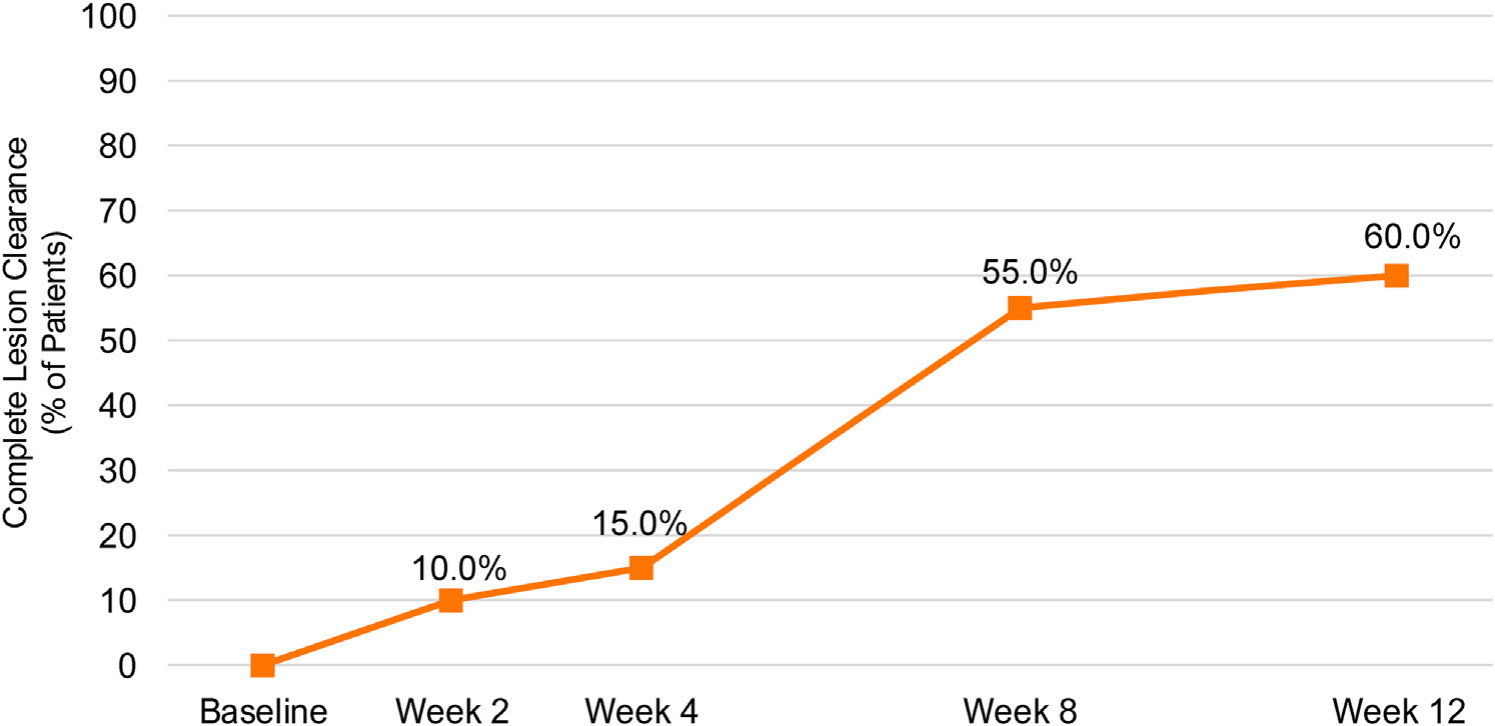
Molluscum contagiosum. Percentage of patients treated with berdazimer gel, 10.3% (*N* = 20) who had complete clearance of all molluscum contagiosum lesions.

**Fig 2:**
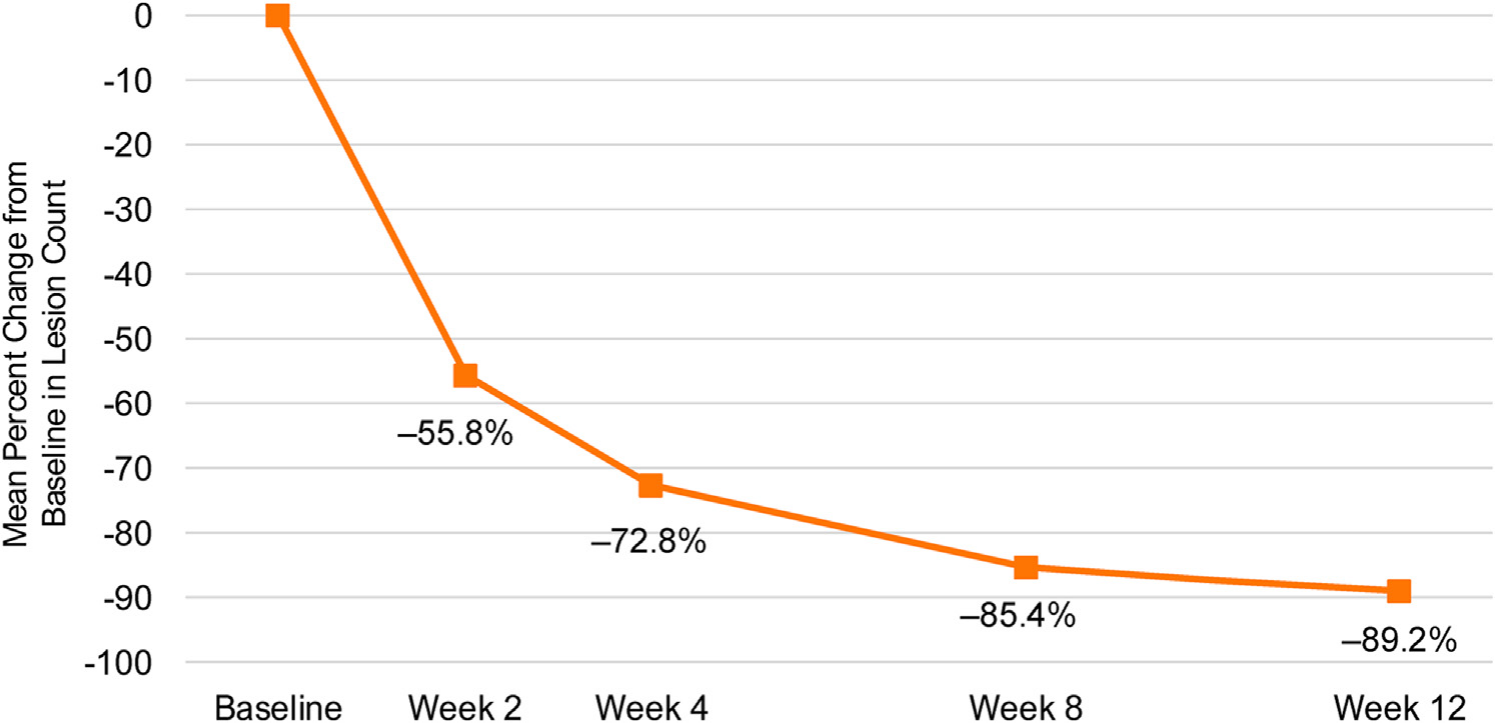
Molluscum contagiosum. Percent change in molluscum contagiosum lesion count from baseline in patients treated with berdazimer gel, 10.3% (*N* = 20).

Complete clearance rates increased over time from 10% (2/20) at week 2 to a peak of 60% (12/20) at week 12 (Fig 1). The percent change in MC lesion count from baseline also improved over time and was −89.2% at week 12 (Fig 2).

There were 13 patients (65%) with treatment-related clinically significant LSRs (Table III). Investigators retrospectively designated the LSRs as the BOTE sign, DII, or a mixture of both at the end of each patient’s final visit. Of the clinically significant LSRs, 3 were judged as the BOTE sign: mild application-site dermatitis, moderate application-site dermatitis, moderate application-site erythema.

**Table III:** Treatment-related clinically significant local skin reactions

Local skin reaction	Berdazimer gel, 10.3% *N* = 20 *N* (%)			
	BOTE	MIX	DII	Total
Total	3 (15.0)	6 (30.0)	9 (45.0)	13 (65.0)
Application-site dermatitis	2 (10.0)	3 (15.0)	1 (5.0)	6 (30.0)
Application-site erythema	1 (5.0)	1 (5.0)	4 (20.0)	6 (30.0)
Application-site pain	0	2 (10.0)	3 (15.0)	5 (25.0)
Application-site pruritus	0	2 (10.0)	3 (15.0)	5 (25.0)
Application-site exfoliation	0	0	3 (15.0)	3 (15.0)
Application-site discoloration	0	1 (5.0)	1 (5.0)	2 (10.0)
Application-site dryness	0	0	1 (5.0)	1 (5.0)
Application-site erosion	0	0	1 (5.0)	1 (5.0)
Application-site scab	0	0	1 (5.0)	1 (5.0)
Application-site infection	0	0	1 (5.0)	1 (5.0)

Local skin reactions judged as MIX and DII were treated as adverse events.

*BOTE*, Beginning of the end; *DII*, drug induced irritation; *Mix*, mixture of BOTE and DII.

The clinically significant LSRs judged as the MIX or DII, or treatment-related application-site AEs, were all mild to moderate in severity with the most common being application site-erythema (25%, *n* = 5), pain (25%, *n* = 5), pruritis (25%, *n* = 5), and dermatitis (20%, *n* = 4) (Table IV). Berdazimer gel, 10.3% application was withheld to all/specific lesion(s) on investigator's direction in 55% (11/20) of patients on 1 or more days due to an AE, and 25% (5/20) missed doses prior to week 12. Of these 16 patients, 10 experienced complete clearance prior to or at week 12 (62.5%). Four patients did not have any treatment withheld at any time during the study: of these, 2 experienced complete clearance by week 12.

**Table IV:** Treatment-related application-site adverse events

Adverse event	Berdazimer gel, 10.3% *N* = 20 *N* (%)			
	Mild	Moderate	Severe	Total
Total	4 (20.0)	9 (45.0)	0	13 (65.0)
Application-site erythema	2 (10.0)	3 (15.0)	0	5 (25.0)
Application-site pain	4 (20.0)	1 (5.0)	0	5 (25.0)
Application-site pruritus	4 (20.0)	1 (5.0)	0	5 (25.0)
Application-site dermatitis	0	4 (20.0)	0	4 (20.0)
Application-site exfoliation	1 (5.0)	2 (10.0)	0	3 (15.0)
Application-site discoloration	2 (10.0)	0	0	2 (10.0)
Application-site dryness	1 (5.0)	0	0	1 (5.0)
Application-site erosion	0	1 (5.0)	0	1 (5.0)
Application-site scab	0	1 (5.0)	0	1 (5.0)
Application-site infection	0	1 (5.0)	0	1 (5.0)

**Fig 3:**
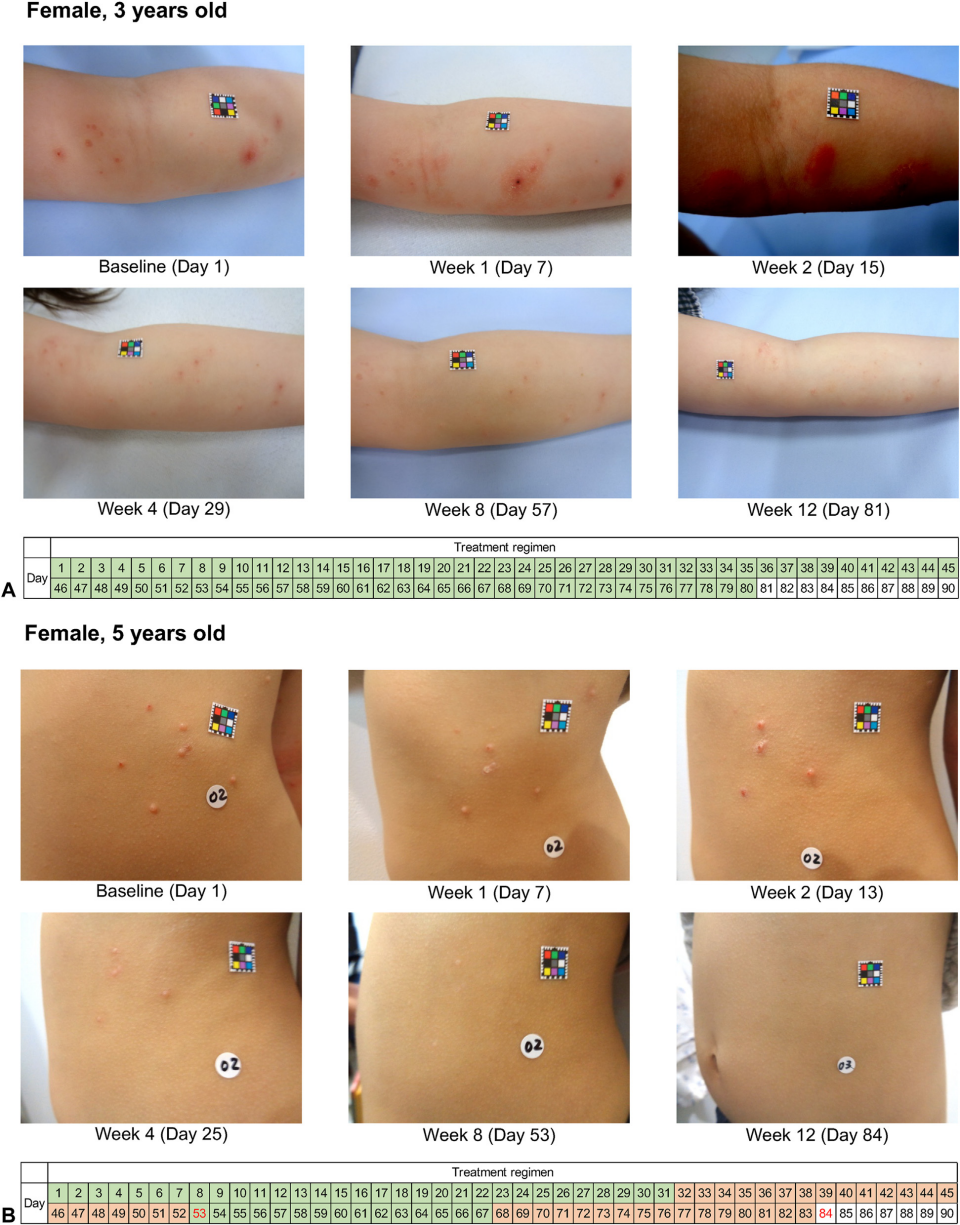

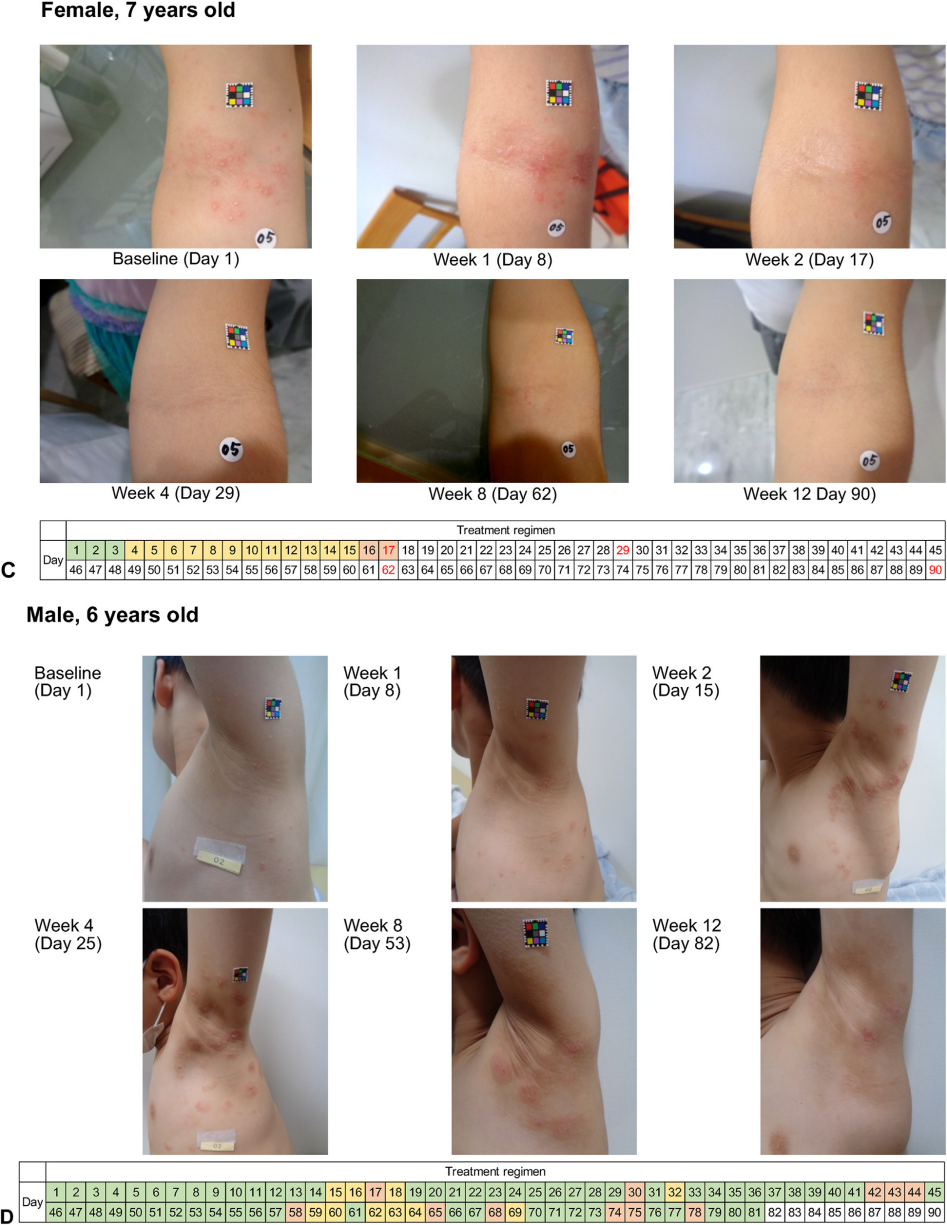
Molluscum contagiosum. Clinical courses of 4 Japanese patients with molluscum contagiosum treated with berdazimer gel, 10.3%. **A,** Arm lesions; (**B**) trunk lesions; (**C**) arm flexural lesions; (**D**) axilla lesions. Bar below photos indicates treatment regimen throughout the study, with *green* representing application to all lesions; *orange* representing application withheld; *yellow* indicating application to specific lesions, and *red* text indicates the day when complete clearance was observed by the investigator.

Photographs of the progression of target lesions of representative patients are shown in Fig 3. No scarring was observed. Two patients experienced mild skin discoloration which appeared to be temporary post-inflammatory hyper- or hypopigmentation, except in 1 patient for whom hyperpigmentation had not yet resolved.

Mean methemoglobin levels at baseline and week 12 were 0.43% and 0.36%, respectively, and were not affected by the application of berdazimer gel, 10.3%.

At week 12, 90% (18/20) of patients were rated as none or mild on the I/S-GSA compared with 100% (20/20) of patients rated as mild or moderate at baseline. In addition, investigators rated 95% (19/20) of patients as very much or much improved on the I-GIC and 75% (17/20) of patients rated themselves as very much or much improved on the S-GIC.

## Discussion

Berdazimer topical gel, 10.3% (SB206 12%) is approved in the United States for topical treatment of MC and is the first prescription medication indicated for MC that can be self-applied by the patient or caregiver. FDA approval was supported by 3 phase 3 studies.^15^^,^^16^ Although the cumulative incidence of MC in Japanese children at 8 years of age is nearly 50%, currently there is neither an established MC standard of care nor an approved MC therapy in Japan.^10^ This study was undertaken to evaluate the safety and tolerability profile of berdazimer gel, 10.3% in Japanese patients with molluscum, as well as exploratory efficacy endpoints.

The safety and tolerability of berdazimer observed in this population was consistent with berdazimer’s favorable profile, with AEs and LSRs consistent in type and severity of previously published studies.^15^^,^^16^^,^^19^ Specifically, mild erythema and pain were the most common AEs in this and previous studies. Also, LSR patterns over time were consistent with previous studies. Because LSRs could be either BOTE, DII, or a mixture of both, the final designation was determined by the investigator retrospectively based on the individual patient’s clinical course. Of note, erythema is a prominent feature of both BOTE and DII and is a common and expected reaction.^13^^,^^18^ AEs and LSRs were followed until resolution. No scarring was observed, and hyper- or hypopigmentation appeared to have been temporary in all but 1 patient based on photographic assessment. Hyperpigmentation in this patient was in the process of resolving.

Application of study medication to all or specific lesion(s) was withheld in 11 of 20 patients (55%) throughout the 12-week study course due to AEs. The majority of patients with drug treatment interruptions in this analysis achieved complete clearance by the end of the study (10/16, 62.5%).

Efficacy was consistent with previous studies in non-Japanese patients, favoring berdazimer.^15^^,^^16^^,^^19^ More than half of the Japanese patients in this study experienced complete clearance of MC lesions at week 12, some experiencing complete clearance as early as week 2 and most before week 12. Berdazimer gel offers advantages over other treatments for MC, because it is a topically applied gel that does not require application in the healthcare provider office.

Limitations of this study include the small sample size and lack of a control comparator group. However, the mean age of patients and number of baseline MC lesions is similar to those in the large phase 3 B-SIMPLE trials supporting FDA approval of berdazimer topical gel.^15^^,^^16^

## Conclusions

Overall, the safety, tolerability, and efficacy profile of berdazimer gel, 10.3% in Japanese patients with MC was favorable and consistent with data from phase 2 and 3 studies conducted in the US. Although a phase 3 study with a larger sample size of Japanese patients with MC will be needed, berdazimer gel, 10.3% holds promise as a potential topical treatment for MC in Japan.

## Conflicts of interest

Dr Kawashima has consultant arrangements with Sato Pharmaceutical Co, Ltd. Authors Tani, Kaneko, Masubuchi, Yasukawa, Sawasaki, and Okada are employees of Sato Pharmaceutical Co, Ltd. Author Enloe and Drs. Geer, Cartwright, and Maeda-Chubachi are employees of Pelthos Therapeutics.
